# Expanding clinical characteristics and genotypic profiling of Yao syndrome in Chinese patients

**DOI:** 10.3389/fimmu.2024.1444542

**Published:** 2024-09-03

**Authors:** Jingyuan Zhang, Xin Huang, Min Shen

**Affiliations:** Department of Rare Diseases, Peking Union Medical College Hospital (PUMCH), Chinese Academy of Medical Sciences & Peking Union Medical College; State Key Laboratory of Complex Severe and Rare Diseases, PUMCH; Department of Rheumatology and Clinical Immunology, PUMCH; National Clinical Research Center for Dermatologic and Immunologic Diseases (NCRC-DID), Ministry of Science & Technology; Key Laboratory of Rheumatology and Clinical Immunology, Ministry of Education, Beijing, China

**Keywords:** nucleotide-binding oligomerization domain containing 2(*NOD2*), systemic autoinflammatory diseases, Yao syndrome, TNF inhibitors, gene variant

## Abstract

**Objectives:**

Yao syndrome (YAOS, OMIM# 617321) is a kind of systemic autoinflammatory diseases (SAIDs) linked to the nucleotide-binding oligomerization domain containing 2 (*NOD2*). Clinical reports of YAOS in China are sparse. Herein, we reported the largest YAOS cohort of Chinese patients to expand the understanding of its phenotype, genotype, and therapeutic responses.

**Methods:**

This study enrolled 15 adult patients diagnosed with YAOS at Peking Union Medical College Hospital from April 2015 to May 2024. Whole-exome sequencing was performed on all patients. Clinical data, genetic variations, and treatment responses were documented and compared with a Caucasian cohort.

**Results:**

The mean age of disease onset was 35 ± 17 years old. The most common clinical manifestations included recurrent high-grade fever (100%), gastrointestinal symptoms (73.3%), arthralgia/arthritis, fatigue, myalgia, and lower extremity swelling (46.7%). All patients exhibited elevated acute-phase reactants during episodes. 12 heterozygous *NOD2* variants were identified, with Q902K in 4 patients, R471C in 3, and variants c.-14C>T, A110T, S127L, R311W, A432V, Y514H, R541P, A661P, K818Q, A886V each found in individual patients. 90% of the patients responded well to glucocorticoids, and 55.6% to sulfasalazine. 66.7% of patients who received TNF inhibitors achieved complete resolution of symptoms. Additionally, one patient each responded favorably to canakinumab and tocilizumab. Compared to the Caucasian cohort, our cohort exhibited a more balanced gender ratio and a higher proportion of recurrent fever, proteinuria/hematuria as well as more frequent leukocytosis, elevated acute phase reactants, and anemia. Lower proportions of arthralgia/arthritis, skin rashes, headaches, and sicca-like symptoms were noted in our cohort. Moreover, a higher proportion of patients in our cohort showed a good response to TNF inhibitors.

**Conclusion:**

Chinese patients with YAOS had more pronounced inflammatory manifestations compared to the Caucasian cohort. Variants c.-14C>T, A110T, S127L, A661P, K818Q, A886V, R471C, and A432V were identified as novel *NOD2* variants in YAOS. TNF, IL-6, and IL-1 inhibitors are the promising treatment options. These findings expand the clinical spectrum, genetic profile, and treatment efficacy of YAOS, underscoring the need for heightened awareness of this disease in diverse populations.

## 1 Introduction

Yao syndrome (YAOS, OMIM# 617321), previously known as nucleotide-binding oligomerization domain containing 2 (*NOD2*)-associated autoinflammatory disorders (NAID), is increasingly acknowledged as a genetically transitional disease (GTD) that intermediate between monogenic and complex polygenic disease (1, 2). To date, known YAOS-associated *NOD2* variants include *NOD2* IVS8^+158^, Arg702Trp (R702W), 1007fs, or Val955Ile (V955I) (3–6), which confer increased susceptibility to YAOS with characteristics of low frequency and low penetrance.

The *NOD2* gene, located at chromosome 16q12-21, encodes NOD2 protein. NOD2 is a member of the NOD-like receptor family that plays an important role in detecting bacterial peptidoglycan and inducing pro-inflammatory, anti-microbial, and antiviral responses, autophagy, and T-cell activation (7–9). It comprises three domains including N-terminal caspase recruitment domains (CARDs), central nucleotide binding and oligomerization domain (NBD), and C-terminal leucine-rich repeats (LRRs) (10, 11). When faced with pathogen-associated molecular patterns (PAMPs), NOD2 self-oligomerizes via their NBD domain to recruit and interact with receptor interaction protein-2 (RIP2), which leads to nuclear factor kappa-B (NF-κB)/mitogen-activated protein kinases (MAPKs) activation and the production of pro-inflammatory cytokines (12). Specific *NOD2* variants are identified as predisposing factors for Crohn’s disease (CD), Blau Syndrome (BS), and YAOS (4, 13, 14).

YAOS was first reported in 2011 by Professor Yao and colleagues (15). The largest cohort study revealed that patients with YAOS are mainly Non-Hispanic White adults with a female predominance (3). It is characterized by recurrent fever, dermatitis, arthritis, swelling of the distal extremities, gastrointestinal and sicca-like symptoms, and eyelid swelling complicated with chronic pain syndrome and even disability (3, 4, 6, 16). Furthermore, it is distinct from CD, BS, and monogenic systemic autoinflammatory diseases (SAIDs) phenotypically and genotypically (17). Spontaneous inflammation has been observed in YAOS, yet the pathogenesis of YAOS remains elusive (5, 18, 19). Therefore, the management of YAOS mainly relies on glucocorticoids and sulfasalazine, and some patients may still experience frequent relapse (16).

YAOS has been predominantly reported in Non-Hispanic White populations (3, 4, 17). Reports of Chinese YAOS patients are sparse (20–22). As awareness among clinicians grows, there is a pressing need to expand the genotypic-phenotypic spectrum associated with YAOS. This study aimed to expand the clinical spectrum and genetic landscape of Chinese patients with YAOS and compare them with those of patients from the Caucasian cohort.

## 2 Methods

### 2.1 Patients

From April 2015 to May 2024, this study enrolled 15 adult patients (≥18 years old) diagnosed with YAOS at our tertiary hospital. YAOS is diagnosed with the fulfillment of 2 major criteria, at least one minor criterion, the molecular criterion, and the exclusion criteria. Major clinical criteria: (1) periodic occurrence≥twice; (2) recurrent fever or dermatitis or both. Minor clinical criteria: (1) Oligo- or polyarthralgia/inflammatory arthritis, or distal extremity swelling; (2) abdominal pain or diarrhea or both; (3) sicca-like symptoms; (4) pericarditis or pleuritis or both. Molecular criterion: *NOD2* IVS8^+158^ or R702W or both, or other rare variants. Exclusion criteria: High titer antinuclear antibodies, inflammatory bowel disease, Blau syndrome, adult sarcoidosis, primary Sjögren syndrome, and monogenic autoinflammatory diseases based primarily on genetic testing results, clinical manifestations, and therapeutic response (e.g., no response to colchicine) (16). Informed consents were obtained from all participants. This study was approved by the Institutional Review Board of Peking Union Medical College Hospital and was performed according to the Declaration of Helsinki.

### 2.2 Statistical analysis

Clinical and demographic information was meticulously documented. Whole-exome sequencing based on the NovaSeq 6000 next-generation sequencing platform was performed in each patient. Sanger sequencing was conducted in probands’ families to identify and confirm *NOD2* sites when feasible. The remission of clinical symptoms was defined as decreases in the duration or frequency of each episode, as well as severity of clinical symptoms after treatment. Response categories were classified as good, partial, or no response, based on the remission of clinical symptoms and normalization of inflammatory markers by more than 80%, 20%–80%, or less than 20%. Clinical data and mutation information were compared with the largest Caucasian cohort (3).

IBM SPSS Statistics (V.25) was used to analyze the data. Continuous variables were expressed as mean ± standard deviation or median and interquartile range. Categorical variables were described as frequency distribution. Student’s t-test was used to compare continuous variables between two patient groups. Chi-square was performed to compare categorical variables. Fisher’s exact test was used to compare the prevalence of different genotypes between our cohort and the general East Asian population. *p*<0.05 was considered statistically significant.

## 3 Results

### 3.1 Baseline demographic data and clinical manifestations of patients with YAOS in our cohort

The demographic and clinical characteristics of Chinese YAOS patients are shown in **Table 1** (details shown in **Supplementary Table 1**). Among these 15 Han patients with YAOS, the gender ratio of male to female was 7: 8, and the average age at the onset of the disease was 35 (_S.D._17) years old, with 2 patients (2/15,13.3%) experiencing disease onset in juvenile (≤14 years old). The average age of disease diagnosis was 42 (_S.D._14) years old, with a median duration of disease at diagnosis was 5 years (ranging from 1 to 30 years). Only 2 patients (1/15,13.3%) reported a family history of recurrent fever and/or autoimmune diseases.

**Table 1:** Summary of the main manifestations and treatments of 15 patients with YAOS in our cohort.

	Variables	Total patients (N=15)
Baseline demographic data	Ratio of gender (M: F)	7:8
	Age at onset, years old	35 ± 17^a^
	Age at diagnosis, years old	42 ± 14^a^
	Disease duration at diagnosis, years	5(1-30)^b^
	Juvenile-onset disease, n (%)	2,13.3%
	Family history, n (%)	2,13.3%
Clinical features, n (%)	Fever	15,100%
	Gastrointestinal symptoms	11,73.3%
	Abdominal pain	8 (8/11,72.7%)
	Diarrhea	7 (7/11,63.6%)
	Constipation	2 (2/11,18.2%)
	Nausea and vomiting	7 (7/11,63.6%)
	Arthralgia/arthritis	7,46.7%
	Poly	7 (7/7,100%)
	Upper extremities	3 (3/7,42.9%)
	Skin rash	5,33.3%
	Periorbital edema	1,6.7%
	Oral ulcers	3,20%
	Myalgia	7,46.7%
	throat pain	3,20%
	Sicca-like symptoms	2,13.3%
	Weight loss	6,40%
	Proteinuria/hematuria	3,20%
	Lymphadenopathy	4,26.7%
	Headaches	4,26.7%
	Chest pain	3,20%
	Pleuritis	2,13.3%
	Lower extremity swelling	7,46.7%
	Fatigue	7,46.7%
Laboratory parameters	Leucocytosis	10,66.7%
	Elevated Ferritin	5 (5/8,62.5%)
	Elevated ESR/CRP	15,100%
	Anemia	10,66.7%
	Elevated TNF	9 (9/10,90%)
	Elevated IL-6	7 (7/11,63.6%)
	Autoantibodies	3 (3/13,23.1%)
Response to treatment	Glucocorticoids	9 GR
	Sulfasalazine	5 GR,1 PR
	NSAIDs	6 PR
	DMARDS	1 PR
	Colchicine	1 GR
	IL-1 inhibitors	1 GR
	TNF-α inhibitors	4 GR,1 PR
	IL-6 inhibitors	1 GR
	Antibiotics	4 PR

YAOS, Yao syndrome; ESR, erythrocyte sedimentation rate; CRP, C-reactive protein; TNF, tumor necrosis factor; IL, interleukin; NSAIDs, nonsteroidal anti-inflammatory drugs; DMARDs, disease-modifying antirheumatic drugs; GR, good response; PR, partial response. ^a^mean ± S.D.; S.D., standard deviation; ^b^median (range).

All patients experienced unprovoked, self-limiting episodes of fevers (Tmax>38.5°C), lasting 1 to 2 weeks and occurring with frequencies ranging from once a week to once a year. 11 patients (11/15, 73.3%) presented with gastrointestinal symptoms, with abdominal pain (8/11,72.7%), diarrhea (7/11,63.6%), nausea and vomiting (7/11,63.6%), and constipation (2/11,18.2%). Additionally, 6 of those with gastrointestinal symptoms reported weight loss, and 4 exhibited anemia, though none had progressive anemia or malnutrition. The abdominal pain usually affects the left and upper abdomen. A history of upper gastrointestinal bleeding was noted in 2 patients. Among 8 patients who displayed abdominal pain, 7 underwent extensive examination including computed tomography (CT), abdominal ultrasound, positron emission tomography (PET)/CT, scanenterography, oesophagogastroduodenoscopy, capsule endoscopy, colonoscopy, and mucous biopsy with no evidence of inflammation bowel disease (IBD).

**Figure 1:**
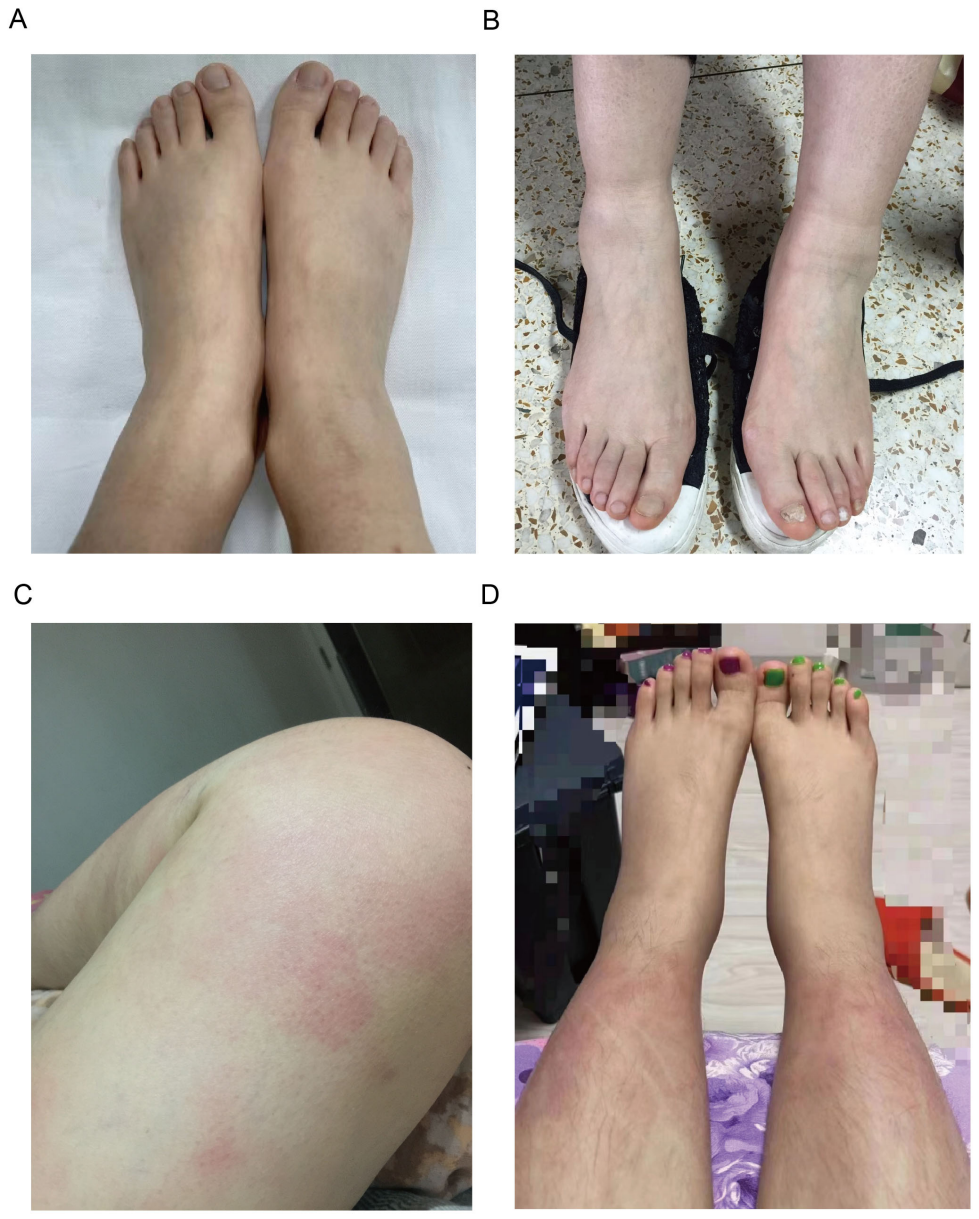
Clinical manifestations of patients with YAOS. **(A)** Representative images of lower extremity swelling of Patient 8. **(B)** Representative images of lower extremity swelling of Patient 4. **(C)** Representative images of skin rashes (erythema) on the thigh of Patient 5. **(D)** Representative images of skin rashes (erythema) on the shank of Patient 11.

Arthralgia/arthritis was observed in 7 patients (7/15, 46.7%), affecting multiple joints across both upper and lower extremities, with 3 (3/7,42.9%) patients involving the upper extremities. 3 patients performed MRI or ultrasound of the joints, 2 presented with joint effusion, and 1 reported bone marrow edema. No bone erosions were reported radiographically. Bilateral lower extremity swelling was noted in 7 patients (7/15,46.7%) (**Figures 1A, B**). Myalgia was reported in 7 patients (7/15, 46.7%), with 2 (2/6,33.3%) only on the legs, and others are generalized. 3 patients underwent further examinations such as MRI, enzymes, electromyography, and muscle biopsies, and showed no typical inflammatory myopathy changes consistent with myositis. Fatigue and lymphadenopathy were also reported in 46.7% of patients.

Skin rash was found in 5 patients (5/15,33.3%), mostly (3/5,60%) manifesting as erythematous patches and plaques, 2 (2/5,40%) as papule, 1 as purpura, and 1 as acne. The rash mainly affected the limbs (4/5,80%) (**Figures 1C, D**), with only 1 on the trunk and face. There were no reports about genital and perianal areas involved, vasculitis, erythema nodosum, or pyoderma gangrenosum.

Headaches occurred in 26.7% of patients. Proteinuria/hematuria, chest pain, periorbital edema, oral ulcers, and throat pain occurred in 20%. Scca-like symptoms (xerostomia), pleuritis, and anxiety and depression were reported in 13.3% of patients. Pericarditis was reported in 6.7%. There were no reports of uveitis.

The acute-phase reactants including erythrocyte sedimentation rate (ESR) and/or C reactive protein (CRP) were increased during episodes in all patients [29 (IQR 51) mm/h, and 78.76 (_S.D._68.2) mg/L, respectively]. Leucocytosis was observed in 10 patients (10/15,66.7%). The mean values for white blood cell (WBC) count, hemoglobin (Hb), and platelets (PLT) were 13.67 (_S.D._6.27)*10^9^/L,115.93g/L (_S.D._23.93), and 266.8 (_S.D._98.18)*10^9^/L, respectively. 6 patients have transient elevation of liver enzymes. Elevated tumor necrosis factor (TNF) was observed in 9 patients (9/10,90%) with a median value of 13.3 (IQR 12.55) pg/mL and interleukin (IL)-6 in 7 patients (7/11,63.6%) with a median value of 10.1 (IQR 57.1) pg/mL. Ferritin was elevated in 5 patients (5/8,62.5) with a median value of 363.2 (IQR 708.50) ng/mL (shown in **Table 2**). Most patients tested negative for autoantibodies, except for 3 patients (3/13,23.1%), including 2 patients who exhibited positive antinuclear antibody (ANA) (1:80-1:160, and 1:80, respectively), and 1 had antiphospholipid antibodies. Abnormal immunoglobulin and complement were found in 4 patients (**Supplementary Table 2**). It suggests that patients with YAOS have a robust inflammatory response at the onset of the disease.

**Table 2:** Summary of the laboratory results of 15 patients with YAOS in our cohort.

Laboratory test	Total patients (N=15)	Reference value
WBC count (×10^9^/L)	13.67 ± 6.27	3.5-9.5
Neutrophils count (×10^9^/L)	8.97 ± 5.75	2.0-7.50
Platelets (×10^9^/L)	266.8 ± 98.18	100-350
Hemoglobin (g/L)	115.93 ± 23.93	120-160
CRP (mg/L)	78.76 ± 68.20	<8
Hs-CRP (mg/L)	75.71(194.6)^a^	<8
ESR (mm/h)	29(51)^a^	0-15
TNF (pg/mL)	13.3(12.55)^a^	<8.1
IL-6 (pg/mL)	10.1(57.1)^a^	<5.9
Ferritin (ng/mL)	363.2(708.5)^a^	14-307

WBC, White blood cell; CRP, C-reactive protein; hs-CRP, hypersensitive C-reactive protein; ESR, erythrocyte sedimentation rate; TNF, tumor necrosis factor; IL, interleukin; ^a^median (IQR).

### 3.2 Genetic features of patients with YAOS in our cohort

**Figure 2:**
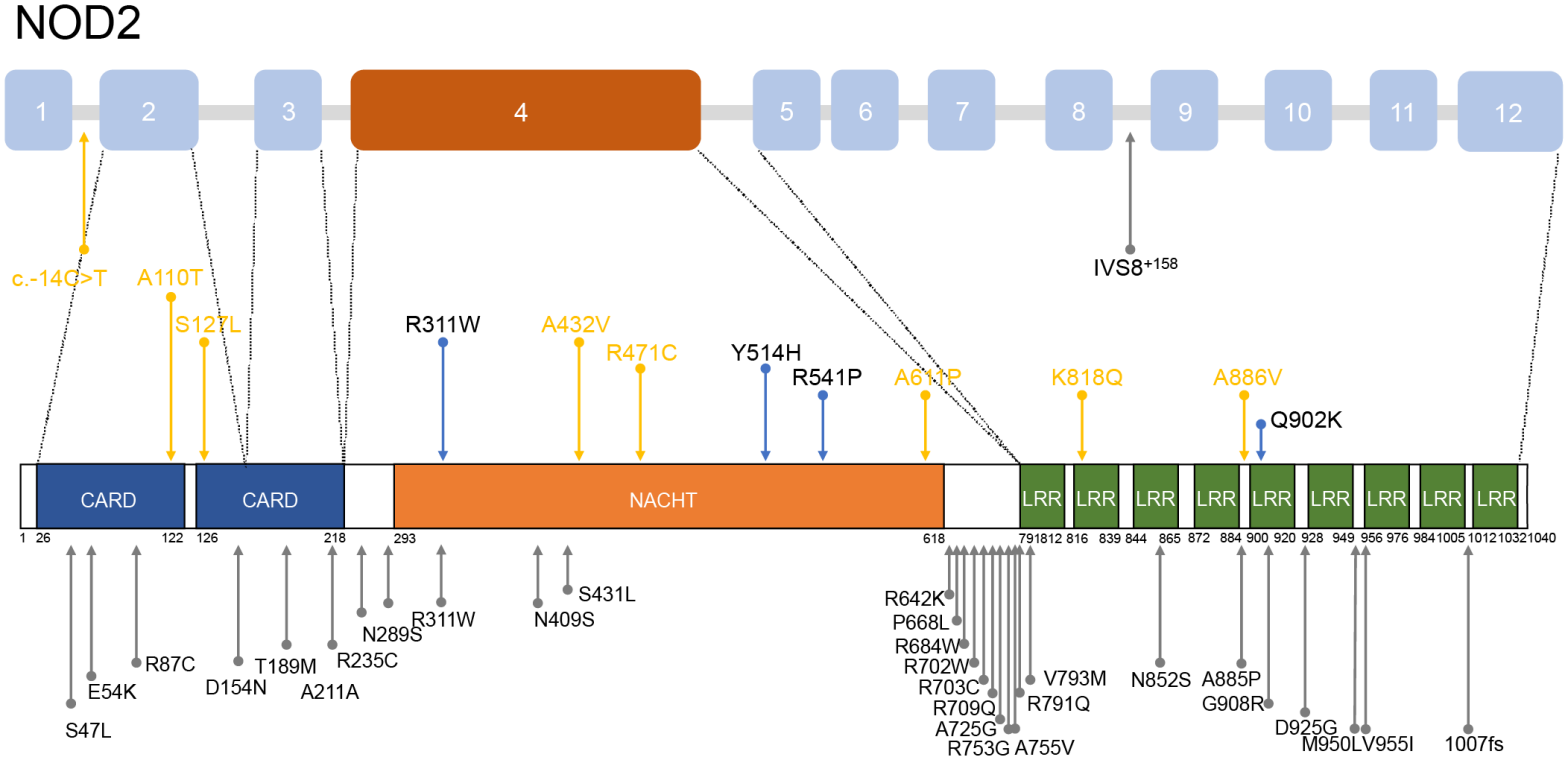
Distribution of *NOD2* variants mentioned in patients with YAOS. Above is a schematic of the *NOD2* gene and below is a schematic of coding regions of the NOD2 protein. The blue arrows denote the variants found in our cohort. The yellow arrows show the novel variants found in our cohort. The gray arrows indicate variants found in other cohorts.

12 heterozygous *NOD2* variants were detected including Q902K in 4 patients, R471C in 3, and single of c.-14C>T, A110T, S127L, R311W, A432V, Y514H, R541P, A661P, K818Q, A886V (**Figure 2**). Additionally, 2 patients carried compound heterozygous involving variants Q902K and R471C as well as R471C and c.-14C>T. Besides, other than *NOD2*, 4 patients carried *MEFV* variants, including 3 with the E148Q variant and one each with R202Q and L110P, which were all considered benign. All the gene variants were rare among the East Asian population, with an allele frequency of less than 0.02 based on a database of Genome Aggregation Database (gnomAD), Exome Aggregation Consortium (ExAC), dPSNP, and 1000 Genomes Project (China). Allele frequencies of *NOD2* variants found in our cohort were significantly elevated in our patients in comparison with healthy individuals (23) and the general East Asian population, except for variants R311W and c.-14C>T. According to the American College of Medical Genetics and Genomics (ACMG) guideline (24), 6 variants were categorized as Variant of Uncertain Significance (VUS), three as likely benign or benign, with the most frequent variants Q902K and R471C classifying as likely benign and benign respectively. c.-14C>T, A110T, S127L, A661P, K818Q, A886V, R471C, and A432V were identified as novel variants in YAOS. Out of the 15 patients with YAOS, families of 11 underwent whole exome sequencing (WES) to detect *NOD2* variants, and 9 were found to be inherited from their parents without *de novo* variants. Details of *NOD2* variants in 15 patients with YAOS are listed in **Table 3**.

**Table 3:** Summary of *NOD2* variants in 15 patients with YAOS.

*NOD2* variants^#^	Location	ACMG	Allele frequency in our cohort (%)	Allele Frequency in Healthy Individuals (%) (23)	Allele frequency in East Asian population (%)	*P* value*
c.2704C>A,p.Q902K	exon 7	Likely Benign	13.3%	2%	0.6127%	0.000
c.1411C>T,p.R471C	exon 4	Benign	10%	3%	1.442%	0.009
c.1622G>C,p.R541P	exon 4	VUS	3.3%	NR	0.1044%	0.034
c.1540T>C,p.Y514H	exon 4	VUS	3.3%	NR	0.0116%	0.007
c.2452A>C,p.K818Q	exon 4	VUS	3.3%	NR	NR	–
c.380C>T,p.S127L	exon 2	Likely Benign	3.3%	NR	0.0233%	0.010
c.2657C>T,p.A886V	exon 7	VUS	3.3%	NR	NR	–
c.1295C>T,p.A432V	exon 4	VUS	3.3%	NR	0.0232%	0.010
c.931C>T,p.R311W	exon 4	Benign	3.3%	0.2%	0.1735%	0.054
c.1981G>C,p.A661P	exon 4	VUS	3.3%	0.6%	0.1043%	0.034
c.328G>A,p.A110T	exon 2	Likely Benign	3.3%	NR	0.1046%	0.034
c.-14C>T	–	Benign	3.3%	NR	0.3750%	0.137

^#^All variants are heterozygous. *NOD2*, nucleotide-binding oligomerization domain containing 2; ACMG, American College of Medical Genetics and Genomics; VUS, variant of uncertain significance; NR, not reported; *comparison of prevalence of different genotypes between our cohort and the general East Asian population, two-sided Fisher’s exact test.

### 3.3 Treatment and outcome

In this study, among the patients who underwent timely follow-up, the median duration of follow-up was 6 (IQR 15) months, and the median interval between visits was 4 (IQR 3) months. As for the treatment, most patients received a combination therapy of glucocorticoids, disease-modifying anti-rheumatic drugs (DMARDs), and/or sulfasalazine. Only 3 patients were once treated with sulfasalazine alone. Overall, 9 patients with YAOS responded well to prednisone (9/10,90%) which showed a good response in fever, gastrointestinal symptoms, arthralgia, and normalization of inflammatory indicators. 5 patients responded well to sulfasalazine (5/9,55.6%) and 1 had a partial clinical response. Oral sulfasalazine contributes to tapering down the dose of glucocorticoids, symptom improvement (fever, gastrointestinal symptoms, rash, lower extremity swelling, and arthritis), extending the interval between flare-ups, and decreasing the severity of attacks. One patient reported significant recovery in her physical functionality and resumed her work. One patient reported adverse reactions including nausea and vomiting and one experienced thrombocytopenia. 3 patients discontinued sulfasalazine due to drug allergies or intolerance. Additionally, most patients were treated with colchicine (7/9,77.8%), and antibiotics (5/8,62.5%) or underwent tonsillectomy without clinical response.

For frequent disease flares, steroid dependence, or no response to multiple therapies, 6 patients were given tumor necrosis factor (TNF) inhibitors, with 4 (4/6, 66.7%) of these patients experiencing a resolution of symptoms (fever, myalgia, and abdominal pain) and a reduction in attack frequency. Canakinumab was effective in 1 patient (n=1/1,100%) for successful tailoring of glucocorticoids. Tocilizumab was effective in 1 patient (n=1/2,50%) for resolution of fever and myalgia. Biologic agents contributed to tapering down the dose of glucocorticoids, reducing attack frequency, improving symptoms, and normalizing inflammatory markers (**Table 1**). 6 patients have a partial response to nonsteroidal anti-inflammatory drugs (NSAIDs) which mainly helped normalize the temperature and alleviate chills but did not alter the interval between disease attacks.

**Figure 3:**
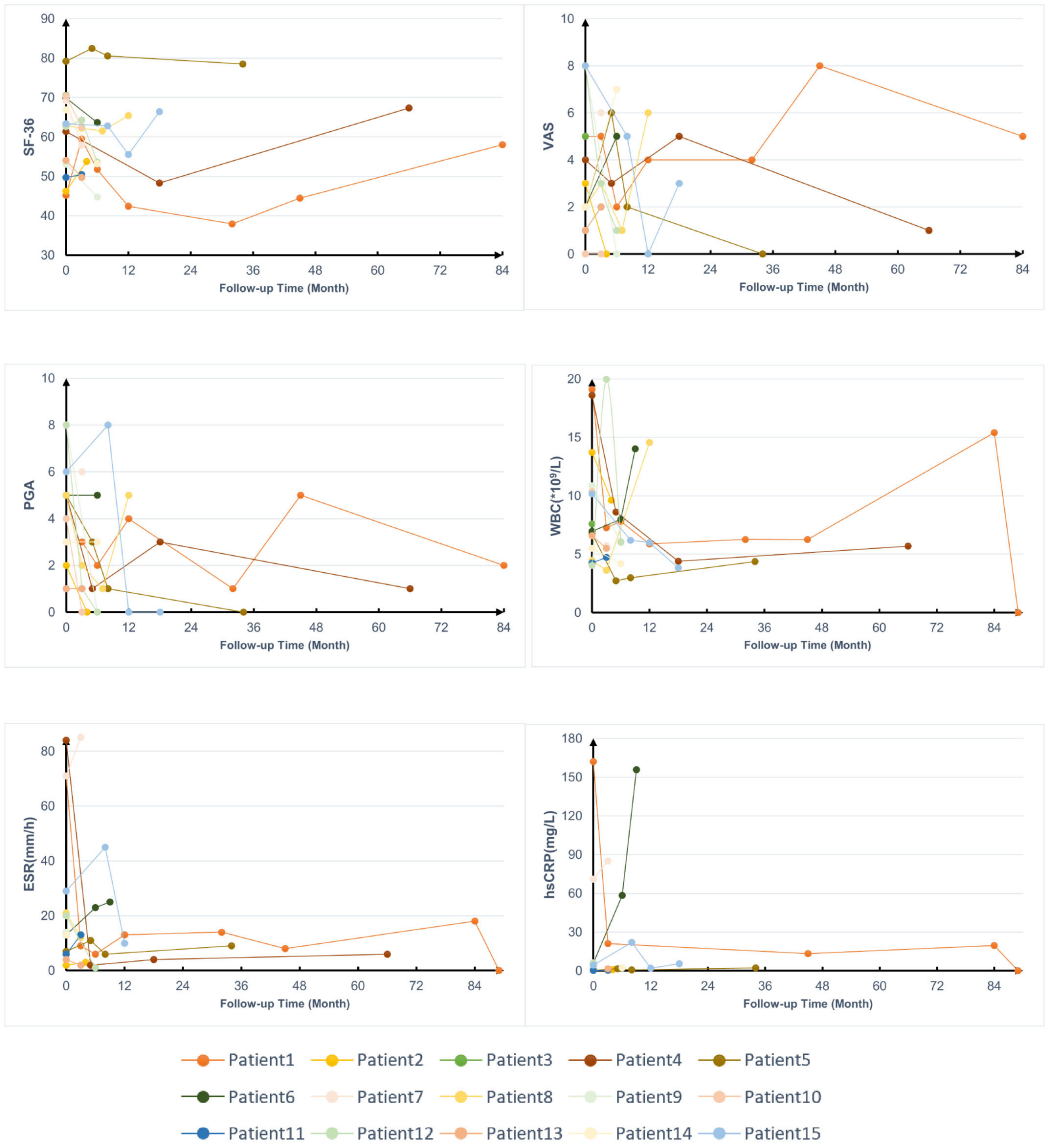
Follow-up data on the treatment of patients with YAOS. SF-36, 36-item Short Form; VAS, visual analog scale; PGA, physician global assessment; WBC, white blood cells; hs-CRP, hypersensitive C-reactive protein; ESR, erythrocyte sedimentation rate.

The average levels of acute-phase reactants, and scores of visual analog scale (VAS), physician global assessment (PGA), and 36-item short-form survey (SF-36) during follow-ups were collected to assess disease activity (**Figure 3**), except for 2 patients who were lost to follow-up, 1 patient didn’t receive treatment after enrollment. Most patients experienced varying degrees of relapse during the follow-up, but after treatment, their SF-36, VAS, and PGA scores as well as WBC, hypersensitive C-reactive protein (hs-CRP), and ESR, showed improvement to some extent.

### 3.4 Phenotype–genotype interaction

Among the 4 patients carrying the Q902K variant, all exhibited lower extremity swelling. This differed from patients carrying other variants (n = 11), where the proportion of lower extremity swelling was 27.3%. In the case of the R471C variant, which was identified in 3 patients, 2 of 3 (66.7%) suffered skin rashes involving limbs, compared to 25% in patients with other variants. Furthermore, *MEFV* was the most frequent co-existing gene. Patients with combined *NOD2* and *MEFV* variants were identified in 4 patients who experienced a higher proportion of headaches (75% vs. 9.1%), chest pain (50% vs. 9.1%), myalgia (75% vs. 36.4%), and weight loss (50% vs. 36.4%). It suggested that *MEFV* may play a modifier role in disease phenotypes rather than acting as a causative gene, supported by no response to colchicine in these patients.

### 3.5 Comparison between the Chinese cohort and the Caucasian cohort

To date, the largest cohort of YAOS was reported in 2015 (3), which enrolled 54 patients who were all Non-Jewish White. This research found that IVS8^+158^ was the most frequent mutation (85%, n = 46). Other common variants included R702W (n = 15,28%), and rare variants (12, 22%). In our cohort, The most common variant was Q902K (n = 4, 26.7%). The gender ratio of male to female in our Chinese patients was nearly balanced, compared to a female predominance in the Caucasian cohort (7:8 vs. 17:37). The average age at disease onset was comparable between our cohort and the Caucasian cohort (35 vs. 33.5 years old). Moreover, the mean disease duration at diagnosis was shorter in our cohort (5 vs. 10.7 years). In terms of symptoms, recurrent fever (100% vs. 61%, *p*<0.05) was more common in our cohort than the Caucasian cohort, as well as poly arthralgia/arthritis (100% vs. 68%), lower extremity swelling (46.7% vs. 32%), myalgia (46.7%% vs. 35%), lymphadenopathy (26.7% vs. 9%), sore throat (20% vs. 5%). Our cohort also reported occurrences of proteinuria/hematuria (3, 21.4%) which was not reported in the Caucasian cohort. One patient is concurrent with nephrotic syndrome and exhibited unexplained intermittent gross hematuria. One patient experienced microscopic hematuria and one patient reported proteinuria. However, frequencies of arthralgia/arthritis (46.7% vs. 87%, *p*<0.05), skin rashes (33.3% vs. 91%, *p*<0.05), headaches (26.7% vs. 56%, *p*<0.05), and sicca-like symptoms (13.3% vs. 56%, *p*<0.05) were significantly less prevalent in our cohort. The frequencies of gastrointestinal symptoms, oral ulcers, chest pain, pleuritis and/or pericarditis, periorbital edema, and weight loss were similar between the two cohorts. In laboratory data, leucocytosis (66.7% vs. 14%, *p*<0.05), elevated acute phase reactants (100% vs. 39%, *p*<0.05), and anemia (66.7% vs. 15%, *p*<0.05) were significantly more common in our cohort (**Table 4**). Concerning treatment response, a greater proportion of patients in our cohort responded with show good response to TNF inhibitors (66.7% vs. 1 partial response), but less to sulfasalazine (55.6% vs. 100%).

**Table 4:** Comparison of the main clinical data and treatments between Chinese and non-Jewish patients.

	Variables	Our cohort (N=15)	Caucasian cohort (3) (n=54)	*P* value*
Baseline demographic data	Ratio of gender (M: F)	7:8	17:37	0.275
	Age at onset, mean ± S.D., years old	35 ± 17	33.5 ± 14.2	0.73
	Age at diagnosis, mean ± S.D., years old	42 ± 14	44.5 ± 14.2	0.547
	Family history, n (%)	2,13.3%	4,7%	0.839
Clinical features, n (%)	Fever	15,100%	33,61%	**0.01**
	Gastrointestinal symptoms	11,73.3%	39,72%	1.000
	Arthralgia/arthritis	7,46.7%	47,87%	**0.003**
	Poly	7 (7/7,100%)	32 (32/47,68%)	0.191
	Upper extremities	3 (3/7,42.9%)	26 (26/47,55%)	0.833
	Skin rash	5,33.3%	49,91%	**0.000**
	Periorbital edema	1,6.7%	1,1.9%	0.390
	Pleuritis	2,13.3%	5,9.3%	1.000
	Pericarditis	0,0%		
	Oral ulcers	3,20%	14,26%	0.895
	Myalgia	7,46.7%	19,35%	0.417
	throat pain	3,20%	3,5%	0.216
	Sicca-like symptoms	2,13.3%	30,56%	**0.004**
	Weight loss	6,40%	22, 41%	0.959
	Proteinuria/hematuria	3,20%	0,0%	**0.009**
	Lymphadenopathy	4,26.7%	5,9%	0.181
	Headaches	4,26.7%	30,56%	**0.048**
	Chest pain	3,20%	9,17%	1.000
	Lower extremity swelling	7,46.7%	17,32%	0.275
Laboratory parameters	Leucocytosis, n (%)	10,66.7%	7,14%	**0.000**
	Elevated ESR/CRP, n (%)	15,100%	21,39%	**0.000**
	Anemia, n (%)	10,66.7%	8,15%	**0.000**
	Autoantibodies, n (%)	3 (3/13,23.1%)	4,7%	0.249
*NOD2* variants, n (%)	Q902K (4,26.7%) R471C (3,20%) Other variants (10, 66.7%)		IVS8^+158^ (46, 85%) R702W (15, 28%) Rare variants (12, 22%)	
Good response to treatment, n (%)	Glucocorticoids	9 (9/10,90%)	21 (21/21,100%)	0.323
	Sulfasalazine	5 (5/9,55.6%)	18 (18/18,100%)	**0.013**
	IL-1 inhibitors	1 (1/1,100%)	1 (1/1,100%)	–
	TNF-α inhibitors	4 (4/6,66.7%)	0 (0/1,0%)	0.429
	IL-6 inhibitors	1 (1/2,50%)	0 (0/1,0%)	1.000

YAOS, Yao syndrome; S.D., standard deviation; ESR, erythrocyte sedimentation rate; CRP, C-reactive protein; TNF, tumor necrosis factor; IL, interleukin; * two-sided Chi-square test or Fisher’s exact test.

Bold indicates p<0.05.

## 4 Discussions

YAOS is a recently recognized, rare autoinflammatory disorder with an estimated population prevalence of 1-10/100000 (3). Our understanding of its clinical profile and etiopathogenesis of YAOS is still in progress (5, 18, 19). As it predominantly occurs in the Caucasian populations, the awareness and knowledge of YAOS in China remain inadequate. To date, there have been only three case reports of YAOS in Chinese patients (20–22), with the first report from our team in 2018 (20). Thus, this underscores the need to expand the clinical features and genetic spectrum of YAOS in China.

Our study revealed that Chinese patients with YAOS have distinct phenotypes compared to Non-Hispanic White. In 2018, three Chinese patients with YAOS lacked skin rashes (20), but our current report shows 33.3% of patients present with skin rashes. When compared with the largest cohort of non-Jewish Caucasian patients (3), we observed a similar average age at disease onset, as well as the proportion of gastrointestinal symptoms, oral ulcers, chest pain, pleuritis and/or pericarditis, periorbital edema, and weight loss. However, the gender ratio is almost equal in our cohort, unlike the female predominance in the Caucasian cohort. Family history was reported in 16.7% of our patients, similar to about 10% in previous reports, indicating sporadic characteristics (6). Our cohort had a higher proportion of recurrent fever, polyarthralgia/arthritis, lower extremity swelling, myalgia, lymphadenopathy, and sore throat. Meanwhile, lower proportions of arthralgia/arthritis, skin rashes, headaches, and sicca-like symptoms were noted in our cohort. Notably, pericarditis, night sweats, asthma, and hearing loss/decrease were absent in our cohort (6, 17, 25). Proteinuria/hematuria was reported in 20% of our patients but not in the Caucasian cohort (4, 25). Low titers of autoantibodies were observed in 23.1% of patients, as was observed in other patients with autoinflammatory diseases (26–28), higher than in the Caucasian cohort (3). More pronounced inflammatory responses were shown in our cohort, evidenced by higher degrees of fever [>38°C, 100% vs 75% (17)] and increased leukocytosis, elevated acute phase reactants, and anemia.

As shown in **Table 3**, the *NOD2* genotype was more prevalent among patients with YAOS relative to the general East Asian population. The lack of *NOD2* IVS8^+158^, R702W, G908R, or L1007fs in Asian populations highlights the importance of ethnic comparisons in identifying susceptibility genes. In our cohort, 86.7% carried a single heterozygous variant of *NOD2*, consistent with other cohorts (14, 17). We reported previously unidentified variants such as variant c.-14C>T, A110T, S127L, A661P, K818Q, and A886V (20). They are considered rare with allele frequency of less than 1% in the Asian population. According to the guidelines of ACMG (24), 50% of variants in our cohort are benign or likely benign, with the rest considered VUS. These findings support the notion that low-frequency variants are increasingly identified in late-onset autoinflammatory diseases. Conservative analysis and 3D protein structure predictions suggested that these variants may impact NOD2 function (data not shown). Thus, we also consider these as novel variants in YAOS. Identifying these potentially clinically meaningful variants may prevent diagnostic delays (2). T helper (T_H_)17 cell dysfunction may play a role in the pathogenesis of YAOS (18) and CD-associated *NOD2* variants may lead to chronic IL-17-dependent gut inflammation (29). Abnormal lymphocyte subsets were found in some patients (Patient 1, 2, and 11 **Supplementary Table 2**), warranting further functional studies on the NOD2-IL-23-IL-17 pathway.

Most patients with YAOS in other cohorts carried variant IVS8^+158^ resided in the intron 8 splicing region of *NOD2*. Nearly 30% carried variant R702W located in exon 4 or variant 1007fs in exon 11, situated between the NACHT and LRR domains (3, 11, 16, 17, 25, 30). In our cohort, as **Figure 2** shows, 12 *NOD2* variants were identified, with 50% located in exon 4 encoding the NACHT region and 16.7% located in the LRR region. It reflects the genetic heterogeneity in different populations. High-penetrance *NOD2* variants, as seen in BS, contrast with the reduced penetrance in YAOS variants (1, 2, 17, 25), suggesting high penetrance variants may influence phenotypic expression (1, 31). Variants R471C and A432V were previously identified in other autoinflammatory diseases (17) but did not exhibit associated clinical manifestations. NOD2 plays an active role in the immune process to detect bacteria. Disease-associated *NOD2* variants located at different loci may exhibit different NOD2-dependent activity (5, 19). However, functional studies of variant A432V, R471C, and Q902K were unable to promote the NF-κB activation (19, 32, 33). Regional and ethnic factors, combined with NOD2 dysfunction, may contribute to disease through abnormal intestinal barrier function and dysbiosis, similar to CD (29). These aforementioned suggested that *NOD2* variants in YAOS may act more as amplifiers or modifiers of disease expression to increase susceptibility to inflammation synergy with environmental factors, rather than a direct initiator of disease (2, 5). They also serve as diagnostic markers for the disease (25). Further genetic and environmental risk factor detection should be performed in patients, to provide a basis for the diagnosis and treatment of YAOS.

26.7% of patients in our cohort also carried heterozygous for *MEFV* variants, particularly E148Q. Gene dosage or additive role of the coexisting gene variants (34, 35) were also evidenced by compound heterozygous *NOD2* variants leading to earlier disease onset age (16 years old) and more frequent skin rash. Patient 15 exhibited FMF-like features (such as serositis and pleuritis) without response to colchicine, resembling the mixed NLR-associated autoinflammatory Disease (NLR-AID) (25).

Regarding the YAOS treatment, glucocorticoids and sulfasalazine were effective for most patients in our cohort and should be considered first-line treatment options (16). TNF inhibitors were beneficial for steroid-dependent patients or those unresponsive to other DMARDs, especially with elevated TNF levels (Patients 6 and 15), different from the reports that TNF inhibitors may only provide modest or no response (3, 16, 17). IL-6 inhibitors can be a treatment option for patients who failed other immunosuppressive agents and had elevated IL-6 levels (Patients 4), aligning with the previous finding (5). TNF and IL-6 inhibitors may not only help the taper of prednisone but serve as a therapeutic alternative for patients with YAOS who have no access to interleukin-1 inhibitors. In line with the reports (16, 36, 37), IL-1 inhibitor canakinumab was effective for refractory Patient 1 with elevated IL-1β in her supernatants of synovial cells (19). In a word, biologics can be tried for refractory cases, and larger cohorts are needed to validate our findings on efficacy.

Our study provides several clinical implications encompassing clinical awareness, genetic research, and personalized treatment strategies. First, recognizing these phenotypic variations between populations can lead to more accurate and timely diagnoses. Second, our study identified novel, rare, and some compound variants in patients with YAOS, future research can focus on developing genetic panels and improving the interpretation of rare variants to aid clinical diagnosis. Besides, multi-center studies across various ethnic groups will be crucial in identifying universally applicable diagnostic markers and therapeutic targets. Understanding the interplay between multiple variants could lead to more effective treatment plans. Finally, biologics, especially TNF inhibitors, are beneficial for patients without access to IL-1 inhibitors. Larger cohorts may help validate these findings and explore the long-term efficacy and safety of these biologics.

The study benefits from uniformity and standardization of the study population but is limited by a small sample size due to the rarity of diseases. Our sample size may not be sufficient to address confounders and only describe notable traits of the disease based on the descriptive nature of this study. Moreover, our study only enrolled Chinese participants and did not cover all regions of China, limiting our ability to investigate the geographic or ethnic differences in clinical manifestations. Therefore, it is necessary to conduct further multi-center and larger-sample prospective studies to complement and evaluate our findings. Second, including whole gene sequencing could identify novel *NOD2* variants in intronic regions potentially missed by current screening methods. Finally, the follow-up times of our cohort are still inadequate. Disease natural history, treatment compliance, and possible long-term inefficacy of biologics have not been fully considered. Future longitudinal studies are needed to collect the long-term impact of various therapeutic interventions.

## 5 Conclusions

Herein, we described the largest case series of Chinese adult patients with YAOS exhibiting more pronounced inflammatory manifestations. 8 variants including c.-14C>T, A110T, S127L, A661P, K818Q, A886V, R471C, and A432V were identified as novel variants in YAOS. TNF, IL-6, and IL-1 inhibitors are the promising treatment options for YAOS. The identification of different phenotypes and novel variants enriches the clinical landscape of YAOS and underscores the need to confirm the pathogenic roles of these variants in larger case-control studies and more functional experiments.

## Data Availability

The original contributions presented in the study are included in the article/**Supplementary Material**. Further inquiries can be directed to the corresponding author.
