# Supplementary material for: Expanding clinical characteristics and genotypic profiling of Yao syndrome in Chinese patients
Source: Front Immunol. 2024 Sep 3;15:1444542. doi: 10.3389/fimmu.2024.1444542 (PMC11406172; doi:10.3389/fimmu.2024.1444542)
Supplement: Supplementary file 1 [file DataSheet1.docx]

Supplementary Material

Expanding Clinical Characteristics and Genotypic Profiling of Yao Syndrome in Chinese Patients

**Jingyuan Zhang^1†^,** **Xin Huang^1†^, Min Shen^1#^**

*** Correspondence:** Min Shen: [shenmpumch@163.com](mailto:shenmpumch@163.com)

# Supplementary Table

| **Supplementary Table 1 Details of Demographic and clinical characteristics of 15 patients with YAOS** | | | | | | | | | | | | | | |  |  |
| --- | --- | --- | --- | --- | --- | --- | --- | --- | --- | --- | --- | --- | --- | --- | --- | --- |
|  | 1 | 2 | 3 | 4 | 5 | 6 | 7 | 8 | 9 | 10 | 11 | 12 | 13 | 14 | 15 |  |
| Ethnicity | Han | Han | Han | Han | Han | Han | Han | Han | Han | Han | Han | Han | Han | Han | Han |  |
| Age at onset, years old | 39 | 9 | 42 | 46 | 20 | 12 | 44 | 18 | 44 | 18 | 24 | 47 | 67 | 49 | 48 |  |
| Age at diagnosis, years old | 49 | 39 | 45 | 47 | 37 | 18 | 47 | 24 | 45 | 27 | 25 | 51 | 72 | 52 | 55 |  |
| Fever | + | + | + | + | + | + | + | + | + | + | + | + | + | + | + |  |
| Tmax | 42℃ | 40℃ | 41℃ | 39.9℃ | 40℃ | 39.2℃ | 39℃ | 38.5℃ | 39℃ | 39℃ | 40℃ | 38.5℃ | 40℃ | 39℃ | 38.6℃ |  |
| Fever duration, days | 7-14 | 2-4 | 4-7 | 2-4 | 5 | 3-4 | 14 | 3-7 | 3-10 | 4-10 | 15 | 7-11 | 7 | 3-5 | 3 |  |
| Fever frequency | once/2-3months | once/1week-1month | once/1-2months | once/4days-1month | once-twice/year or 2-3times/month | once/20days | once/2weeks or 2-3months | once/1-1.5months | once/2month | once/1-2month | once-twice/year | once/20days | once/10days  or 2-3months | once/1-2weeks or once-twice/2-3months | once/10days or three-four times/per year |  |
| Gastrointestinal symptoms | + | - | + | - | + | - | - | + | + | + | + | + | + | + | + |  |
| Abdominal pain | + | - | + | - | - | - | - | + | + | + | - | + | - | + | + |  |
| Abdominal pain Location | Left upper abdomen | - | Bilateral abdomen | - | - | - | - | Left lower abdomen | Middle and left side of the abdomen | Upper abdomen | - | Upper and left upper abdomen | - | Lower abdomen | Around the umbilicus |  |
| constipation | - | - | - | - | - | - | - | + | + | - | - | - | - | - | - |  |
| Diarrhea | + | - | - | - | - | - | - | - | - | + | + | + | + | + | + |  |
| Nausea and vomiting | + | - | - | - | + | - | - | - | + | - | + | + | + | - | + |  |
| Arthralgia/arthritis | + | - | + | + | + | - | - | + | + | - | - | - | + | - | - |  |
| Oligo | - | - | - | - | - | - | - | - | + | - | - | - | - | - | - |  |
| Poly | + | - | + | + | + | - | - | + | - | - | - | - | + | - | - |  |
| Upper extremities | + | - | - | + | - | - | - | - | + | - | - | - | + | - | - |  |
| Skin rash | - | - | - | - | + | + | - | - | - | - | + | - | + | + | - |  |
| Patches/plaques | - | - | - | - | + | - | - | - | - | - | + | - | + | - | - |  |
| Papule | - | - | - | - | - | + | - | - | - | - | + |  | - | - | - |  |
| Acne | - | - | - | - | - | - | - | - | - | - | - | - | - | + | - |  |
| Purpura | - | - | - | - | - | - | - | - | - | - | - | - | + | - | - |  |
| Limbs | - | - | - | - | + | + | - | - | - | - | + |  | + | - | - |  |
| Trunk | - | - | - | - | - | - | - | - | - | - | - | - | - | + | - |  |
| Face | - | - | - | - | - | - | - | - | - | - | - | - | - | + | - |  |
| Periorbital edema | - | - | + | - | - | - | - | - | - | - | - | - | - | - | - |  |
| Pleuritis | + | - | - | - | - | - | - | - | - | - | - | - | - | - | + |  |
| Pericarditis | - | - | - | - | - | - | - | - | - | - | - | - | - | - | - |  |
| Oral ulcers | - | + | - | - | - | - | - | - | - | + | - | - | - | + | - |  |
| Myalgia | - | - | - | + | - | + | + | + | - | - | + | - | - | + | + |  |
| throat pain | - | + | - | - | - | - | + | + | - | - | - | - | - | - | - |  |
| Sicca-like symptoms | - | + | - | - | - | - | + | - | - | + | - | - | - | - | - |  |
| Weight loss | + | - | - | - | - | - | - | - | + | + | + | + | - | + | - |  |
| Lymphadenopathy | - | + | - | - | - | - | - | - | + | + | + | - | - | - | + |  |
| Headaches | - | - | - | - | - | - | + | - | - | - | - | - | + | + | + |  |
| Chest pain | - | - | - | - | - | - | + | + | - | - | - | - | - | + | - |  |
| Lower extremity swelling | + | - | - | + | + | - | + | + | - | - | + | - | - | + | - |  |
| Anxiety and depression | + | - | - | - | - | - | - | + | - | - | - | - | - | - | - |  |
| Fatigue | + | + | + | - | - | - | + | - | - | - | + | - | + | - | + |  |
| Proteinuria/hematuria | - | + | - | - | - | - | + | - | - | + | - | - | - | - | - |  |
| Other conditions | - | - | - | - | - | Parotitis | - | Tonsillitis | - | - | Photosensitivity | - | Interstitial lung disease | - | Serositis |  |
| Family history | - | - | - | - | - | - | - | - | - | - | - | + | - | - | + |  |
| Infection | Salmonella | - | - | - | - | - | - | - | - | - | - | Fungal esophagitis | T-SPOT+,Mycoplasma Escherichia coli,Candida parapsilosis | Viruses, Mycoplasma | - |  |
| Glucocorticoids | GR | GR | GR | GR | GR | NR | GR | - | - | - | - | GR | GR | GR | GR |  |
| Sulfasalazine | PR | - | GR | Allergy | GR | - | Allergy | GR | NR | GR, Nausea, and Vomiting | - | - | - | GR, Thrombocytopenia | - |  |
| NSAIDs | PR | - | - | - | - | PR | - | PR | - | - | PR | - | - | PR | PR |  |
| Other DMARDs | NR | - | - | NR | - | NR | - | - | NR | - | - | - | PR | Thrombocytopenia | - |  |
| Colchicine | - | - | - | NR | - | NR | NR | NR | NR | - | - | NR | GR | Intolerance | NR |  |
| IL-1 inhibitors | GR | - | - | - | - | - | - | - | - | - | - | - | - | - | - |  |
| TNF-α inhibitors | PR(Etanercept) NR(Adalimumab) | - | - | NR | - | GR(Etanercept) | - | - | GR(Adalimumab) | - | - | GR(Adalimumab) | - | - | GR(Adalimumab) |  |
| IL-6 inhibitors | NR | - | - | GR | - | - | - | - | - | - | - | - | - | - | - |  |
| JAK inhibitors | NR | - | - | Vomiting | - | - | - | - | - | - | - | - | - | - | - |  |
| Tonsillectomy | - | NR | - | - | - | - | - | NR | - | - | - | - | - | - | - |  |
| Antibiotics | NR | - | - | - | - | - | NR | - | NR | - | NR | PR | PR | PR | PR(Clarithromycin) NR |  |
| *NOD2 variants* | c.2704C>A,p.Q902K | c.1622G>C,p.R541P | c.1540T>C,p.Y514H | c.2452A>C,p.K818Q | c.2704C>A,p.Q902K;  c.1411C>T,p.R471C | c.1411C>T,p.R471C; c.-14C>T | c.2704C>A,p.Q902K | c.380C>T,p.S127L | c.2657C>T,p.A886V | c.1295C>T,p.A432V | c.931C>T,p.R311W | c.1981G>C,p.A661P | c.328G>A,p.A110T | c.2704C>A,p.Q902K | c.1411C>T,p.R471C |  |
|  |  |  |  |  |  |  |  |  |  |  |  |  |  |  |  |  |
| Other variants | - | - | - | - | - | - | *MEFV* c.605G>A,R202Q | - | *MEFV* c.442G>C*,* E148Q | - | - | - | - | *MEFV* c.442G>C, E148Q *MEFV* c.329T>C,L110P | *MEFV* c.442G>C,p.E148Q |  |
| NSAIDs, nonsteroidal anti-inflammatory drugs; DMARDs, disease-modifying antirheumatic drugs; TNF, tumor necrosis factor; IL, interleukin; JAK, Janus-activated kinase; NOD2, nucleotide-binding oligomerization domain containing 2; GR, good response; PR, partial response; NR, no response. | | | | | | | | | | | | | | | |  |

| **Supplementary Table 2 Laboratory data of the 15 patients with YAOS** | | | | | | | | | | | | | | |  |  |  |
| --- | --- | --- | --- | --- | --- | --- | --- | --- | --- | --- | --- | --- | --- | --- | --- | --- | --- |
|  | 1 | 2 | 3 | 4 | 5 | 6 | 7 | 8 | 9 | 10 | 11 | 12 | 13 | 14 | 15 | Reference value |  |
| WBC (×10^9^/L) | 20.86 | 14.90 | 7.60 | 18.6 | 6.78 | 14.01 | 6.65 | 14.55 | 12.82 | 16.56 | 3.80 | 21.47 | 24.03 | 6.14 | 16.33 | 3.5-9.5 |  |
| NEU (×10^9^/L) | 19.78 | 11.20 | 5.6 | 17.41 | 4.20 | 10.86 | 4.38 | 3.29 | 11.33 | 14.63 | 2.48 | 2.03 | 7.58 | 4.96 | 14.75 | 2.0-7.50 |  |
| PLT (×10^9^/L) | 194 | 267 | 272 | 328 | 203 | 136 | 315 | 371 | 256 | 450 | 317 | 279 | 370 | 86 | 158 | 100-350 |  |
| Hb (g/L) | 77 | 114 | 111 | 102 | 109 | 154 | 136 | 103 | 139 | 162 | 114 | 132 | 93 | 99 | 94 | 120-160 |  |
| ESR (mm/h) | 85 | 21 | 23 | 84 | 11 | 25 | 85 | 21 | 14 | 29 | 36 | 20 | 72 | 62 | 64 | 0-15 |  |
| CRP (mg/L) | 147.3 | 41 | 4.39 | - | - | - | 190.61 | 0.53 | 84.02 | 177.85 | 12.4 | 36.34 | 80 | 92 | - | <8 |  |
| hs-CRP (mg/L) | 211.37 | 0.85 | 1.29 | 64.1 | 1.56 | 155.85 | 217.01 | 0.67 | - | 191 | 6.87 | 6.14 | 87.31 | 154.67 | 257.9 | <8 |  |
| Ferritin (ng/mL) | 1920 | 13 | - | 378.4 | - | 133 | 348 | - | - | - | 171 | - | 578 | 942 | - | 14-307 |  |
| ALT (U/L) | 208 | 12 | 28 | 21 | 8 | 15 | 23 | 14 | 12 | 21 | 12 | 15 | 50 | 19 | 8 | 7-40 |  |
| AST (U/L) | 55 | 19 | 30 | 15 | 14 | - | 14 | 20 | - | 17 | 17 | 18 | 34 | - | 15 | 13-35 |  |
| LDH (U/L) | 742 | 139 | 182 | 250 | 181 | - | 138 | 128 | - | 175 | 212 | 163 | 275 | - | 151 | 0-250 |  |
| TG (mmol/L) | 3.38 | 0.58 | - | 1.37 | - | - | - | 1.12 | 1.95 | - | 1.95 | 1.46 | - | - | 1.63 | 1.45-1.70 |  |
| TNF (pg/mL) | 33.8 | 20.4 | 12.6 | 12.2 | - | 51.8 | - | 14.0 | - | - | 11.5 | - | 8.0 | 10.3 | 20 | <8.1 |  |
| IL-6 (pg/mL) | 192 | 4.8 | 2.8 | 10.1 | - | 11.6 | 42.6 | 2.0 | - | - | 4.96 | - | 9.2 | 61.9 | 110 | <5.9 |  |
| IL-10 (pg/mL) | 5.9 | - | - | - | - | - | - | 5 | - | - | 5 | - | - | 5 | 5 | <9.1 |  |
| IFN-γ (pg/mL) | - | - | - | - | - | - | 32.8 | - | - | - | 2.5 | - | - | - | <8.5 | <16.2 |  |
| IgG (g/L) | 3.3 | 11.7 | 7.42 | 7.11 | 12.32 | 15.45 | 10.7 | 19.46 | - | 9.71 | 14.31 | 8.76 | 9.06 | 9.51 | 8.89 | 7-17 |  |
| IgG1 (mg/L) | 7590 | - | - | - | - | - | - | - | - | - | - | - | 4850 | - | - | 4900-11400 |  |
| IgG4 (mg/L) | 42 | - | - | - | - | - | - | - | - | - | - | - | 380 | - | - | 80-1400 |  |
| IgA (g/L) | 0.55 | 2.18 | 1.48 | 2.65 | 3.29 | 3.2 | 1.46 | 2.65 | - | 3.14 | 2.45 | 2.74 | 1.91 | 1.43 | 1.62 | 0.7-4.0 |  |
| IgM (g/L) | 0.3 | 1.17 | 0.83 | 0.95 | 0.52 | 0.77 | 0.607 | 2.09 | - | 1.12 | 2.70 | 1.01 | 1.04 | 1.20 | 0.63 | 0.4-2.3 |  |
| C3 (g/L) | 0.774 | 0.59 | 1 | 117 | - | 1.269 | - | 0.74 | - | 0.981 | 1.188 | 1.205 | 1.077 | 1.453 | 1.585 | 0.73-1.46 |  |
| C4 (g/L) | 0.095 | 0.163 | 0.236 | 193 | - | 0.19 | - | 0.113 | - | 0.178 | 0.204 | 0.255 | 0.297 | 0.357 | 0.303 | 0.1-0.4 |  |
| RF (IU/mL) | 15.1 | <20 | 14 | - | 1.2 | 3.1 | <11.3 | 699 | - | 2.3 | - | - | 3 | 4.2 | - | 0-20 |  |
| Lymphocyte subsets | B cells,NK cells,T,CD4^+^T,CD8+T,naïve CD4^+^T↓ | CD3^+^CD4^+^T%,CD4^+^/CD8^+^T,CD19+B%↑ CD3^+^CD8^+^T%,NK%↓ | - | - | - | - | - | - | - | - | NK,T,CD8^+^Tcells↓ | - | - | - | - | - |  |
| Other autoantibodies | ANA(+)1:80-1:160, Ro52, Jo-1, AMA-M2 | No | aCL β2GPⅠ | No | No | No | No | - | - | No | No | No | ANA1:80 HLA-B27 | No | No | - |  |
| YAOS, yao syndrome; WBC, white blood cell; NEU, neutrophil; PLT, platelet; Hb, hemoglobin; ESR, erythrocyte sedimentation rate; CRP, C-reactive protein; hs-CRP, hypersensitive C-reactive protein; ALT, alanine aminotransferase; AST, aspartate aminotransferase; LDH, lactate dehydrogenase; TG, triglyceride; TNF, tumor necrosis factor; IL, interleukin; IFN, interferon; Ig, immunoglobulin; RF, rheumatoid factor;-, not done; NK, natural killer; HLA, human leukocyte antigen; ANA, antinuclear antibody; AMA, anti-mitochondrial antibody; aCL, anticardiolipin antibody; β2GPⅠ, anti-beta 2 glycoprotein 1 antibody. | | | | | | | | | | | | | | | | |  |
|  |  |  |  |  |  |  |  |  |  |  |  |  |  |  |  |  |  |
|  |  |  |  |  |  |  |  |  |  |  |  |  |  |  |  |  |  |
